# Meaningful Activity, Psychosocial Wellbeing, and Poverty During COVID-19: A Longitudinal Study

**DOI:** 10.1177/00084174231160950

**Published:** 2023-03-23

**Authors:** Carrie Anne Marshall, Rebecca Gewurtz, Julia Holmes, Brooke Phillips, Suliman Aryobi, Tracy Smith-Carrier

**Keywords:** Mental health, Marginalized groups, Occupation, Occupational deprivation, Social justice, Occupational justice, Occupational therapy, Disability, Health equity, Income insecurity, déprivation occupationnelle, équité en matière de santé, ergothérapie, groupes marginalisés, incapacité, insécurité du revenu, justice occupationnelle, justice sociale, occupation, santé mentale

## Abstract

**Background:** Only a few studies have explored experiences of meaningful activity and associations with psychosocial wellbeing during COVID-19. None reflect a Canadian context or focus on persons living in poverty. **Purpose:** To identify experiences and associations between meaningful activity and psychosocial wellbeing for persons living in poverty during the first year of COVID-19. **Method:** We delivered a quantitative survey at three time points during the first year of the pandemic supplemented by qualitative interviews at Time(T) 1 and 1 year later at T3. **Findings:** One hundred and eight participants completed T1 surveys, and 27 participated in qualitative interviews. Several statistically significant correlations between indices of meaningful activity engagement and psychosocial wellbeing were identified across T1–T3. Meaningful activity decreased from T1–T3 [X^2^ (2, n = 49) = 9.110, *p *< .05], with a significant decline from T2–T3 (z = −3.375, *p *< .001). In T1 qualitative interviews, participants indicated that physical distancing exacerbated exclusion from meaningful activities early in the pandemic. At T3 (1 year later), they described how classist and ableist physical distancing policies layered additional burdens on daily life. **Implications:** Meaningful activity engagement and psychosocial wellbeing are closely associated and need to be accounted for in the development of pandemic policies that affect persons living in low income. Occupational therapists have a key role in pandemic recovery.

## Résumé

**Description.** Quelques études seulement se sont penchées sur les activités significatives et leur lien avec le bien-être psychosocial durant la pandémie de COVID-19. Aucune d’entre elles ne reflète le contexte canadien ou ne se concentre sur les personnes en situation de pauvreté. **But.** Identifier les expériences et les associations entre les activités significatives et le bien-être psychosocial pour les personnes en situation de pauvreté durant la première année de la pandémie de COVID-19. **Méthodologie.** Nous avons réalisé une enquête quantitative à trois moments durant la première année de la pandémie, complétée par des entretiens qualitatifs au moment T1 et un an plus tard, au moment T3. **Résultats.** 108 participants ont répondu aux enquêtes T1 et 27 personnes ont participé aux entretiens qualitatifs. Plusieurs corrélations statistiquement significatives entre les indices d’engagement dans des activités significatives et le bien-être psychosocial ont été identifiées entre T1 et T3. L’activité significative a diminué de T1 à T3 [X^2^ (2, *n* = 49) = 9,110, *p* < 0,05], avec un déclin significatif de T2 à T3 (z = −3,375, *p* < 0,001). Lors des entretiens qualitatifs à T1, les participants ont indiqué que la distanciation physique avait exacerbé l’exclusion des activités significatives au début de la pandémie. À T3 (un an plus tard), ils ont décrit comment les politiques de distanciation physique classistes et capacitistes avaient ajouté au fardeau de la vie quotidienne. **Conséquences.** L’engagement dans des activités significatives et le bien-être psychosocial sont étroitement liés et doivent être pris en compte dans l’élaboration de politiques pandémiques qui affectent les personnes à faible revenu. Les ergothérapeutes ont un rôle clé à jouer dans le rétablissement en lien avec la pandémie.

## Introduction

Physical distancing policies implemented during the COVID-19 pandemic have been essential for controlling viral spread; however, an unintended side effect of these policies is that they have imposed restrictions on meaningful activity and are associated with decreases in psychosocial wellbeing (Cruyt et al., 2021; Tigershtrom & Boyraz, 2021; Wegner et al., 2022). Prior to the pandemic, key population groups experienced systematic exclusion from meaningful activities, including persons living in poverty (Beagan et al., 2018; Hammell, 2015, 2017; Marshall, Boland, et al., 2019). During the pandemic, public health policies may have inadvertently deepened experiences of exclusion for a population whose opportunities for meaningful activity were already limited prior to COVID-19 (Hammell, 2020). While recent studies have identified experiences of meaningful activity engagement and psychosocial wellbeing during the COVID-19 pandemic for youth (Wegner et al., 2022) and the general population (Cruyt et al., 2021; Tigershtrom & Boyraz, 2021); none to our knowledge have explored experiences of meaningful activity for persons living in poverty during this time. This is an essential knowledge for understanding how meaningful activity may be implicated in the health inequities that persons living in poverty have experienced during this historic period (Gravlee, 2020).

### Intersectionality Theory and Meaningful Activity Engagement During COVID-19

Intersectionality theory emerged from feminist thought, and posits that individuals are afforded opportunities and societal privilege through the social locations that they occupy (Crenshaw, 2017). Social locations include race, gender, ability, sexual orientation, and socio-economic status. These social locations accumulate and determine the privileges afforded, or oppression experienced by persons in society. Individuals living in poverty are deeply oppressed not only by having an insufficient income to purchase needed resources and to participate in the economic activities of society, but also by the stigma of poverty itself (Hansen et al., 2014). The oppression faced by women, persons of color, persons living with disabilities, and persons who identify as 2SLGBTQ + places these groups at increased risk of experiencing poverty (Crenshaw, 2017). For this reason, persons living in poverty are frequently oppressed by layers of social locations that serve to limit opportunities for meaningful activity.

### COVID-19, Health Inequities, and Occupational Therapy

The COVID-19 pandemic has exposed pre-existing inequities for persons who live in low income (Benfer et al., 2020). Occupational therapists often support persons living in low income both as practitioners and as advocates (Kirsh, 2015). Examples of such populations include individuals who experience homelessness (Marshall et al., 2021) individuals living with disabilities (Jongbloed, 2016), and tenants who live in social housing (Marshall, Tjörnstrand, et al., 2020). During the COVID-19 pandemic, the mental health of a range of disadvantaged groups including persons living with disabilities (Czeisler et al., 2021), older persons (Steptoe & Di Gessa, 2021), and persons living with mental illness (Yao et al., 2020) have all experienced declines in psychosocial wellbeing. One factor that may be implicated is access to meaningful activity in the context of pandemic restrictions. As occupational therapists are experts in how individuals are able to function and participate in meaningful activities (Egan & Restall, 2022), this topic has particular relevance for the profession. To guide occupational therapy practice and public policy that affect the lives of persons living in poverty during COVID-19 and after, there is a need to identify experiences of meaningful activities, and associations with psychosocial wellbeing for this population during this historic period.

### “Meaningful Activity,” “Boredom,” and How They Relate to Psychosocial WellBeing

**“**Meaningful activities” represent what a person “does” with their time that is meaningful to them personally, professionally, and socially (Egan & Restall, 2022). Time use, on the other hand, is a broad construct that refers to how a person spends their time, including both the activities in which they are engaged, and periods of unoccupied time (Gershuny & Sullivan, 2019). The meaning attributed to one's activities is often derived from fulfilling a life purpose, stimulation, connecting with others, or meeting spiritual needs. While meaningful activities have historically been regarded as solely positive for psychosocial wellbeing in occupational therapy and occupational science, scholars have increasingly recognized that activities that are meaningful may simultaneously impose both negative and positive impacts (Kiepek et al., 2018).

Boredom is inherently related to meaningful activity, and is defined as “the aversive experience of wanting, but being unable to engage in satisfying activity” (Eastwood et al., 2012, p. 482). Boredom emerges from a lack of access to activities that are meaningful, or a lack of meaning or challenge in the activities in which one is able to engage (van Tilburg & Igou, 2012). Individuals living in poverty often experience exclusion from meaningful activities due to living in poverty and lacking social networks that provide access to such activities (Marshall et al., 2022; Marshall, Tjörnstrand, et al., 2020). A lack of access to meaningful activity may further deepen health inequities as the boredom that results can be prolonged and pervasive in the lives of persons living in poverty, and this pattern of boredom has been associated with a range of threats to psychosocial wellbeing in previous research (Biolcati et al., 2018; Elpidorou (2022); Marshall, McIntosh, et al., 2019; Marshall, Roy, et al., 2019; Weissinger, 1995).

### The Current Study

While recent research has focused on how meaningful activity has been disrupted during the COVID-19 pandemic and the ways in which this disruption is associated with indices of psychosocial wellbeing for the general population and youth (Cruyt et al., 2021; Tigershtrom & Boyraz, 2021; Wegner et al., 2022), few studies have explored the specific experiences of persons living in poverty during this time. Understanding the ways in which physical distancing policies influence meaningful activity, specifically for persons living in poverty, is essential both for informing how occupational therapists support individuals in their current practice and to inform practice during future, anticipated pandemics (Kamalakannan & Chakraborty, 2020). Informed by intersectionality theory (Crenshaw, 2017), we conducted this study to fill this gap in existing literature. This research was guided by three related questions: (1) What was the experience of meaningful activity engagement and psychosocial wellbeing for persons living in low income during the first year of the COVID-19 pandemic? (2) How were indices of meaningful activity engagement associated with measures of psychosocial wellbeing? (3) How did indices of meaningful activity engagement and psychosocial wellbeing change over this time period?

## Method

We conducted a concurrent explanatory mixed methods longitudinal study (Creswell & Plano-Clark, 2018). We focused on persons living in “low income” defined according to the low-income measure (LIM) thresholds established by Statistics Canada (2022). The LIM is based on a combination of household income and family composition. Individuals living below the LIM threshold are deemed to be living in a state of poverty, resulting in the inability to meet basic needs (Statistics Canada, 2022). In this study, we followed the same participants living in low income in Ontario, Canada using surveys delivered at three time points, and qualitative interviews delivered at the beginning, and end of the first year of the COVID-19 pandemic.

### Recruitment

After receiving ethics approval from both Western and McMaster Universities, we deployed our Time(T) 1 survey to potential participants. The survey link was shared by email with a range of advocacy groups, social housing organizations, and health and social care organizations in Ontario, Canada who agreed to share the survey with potential participants. We also shared a link to our survey on Twitter and Facebook. We recruited participants in one province only as physical distancing policies were provincially mandated in Canada and varied across the country. Recruiting in one province ensured that participants in our research would be exposed to a similar public health policy environment throughout our study. As our team was primarily situated within the province of Ontario, we chose this province given our familiarity and embeddedness within the policy environment during data collection.

***Inclusion and exclusion criteria:*** We included participants who were (1) over the age of 18; (2) were living in low income according to the 2019 LIM criteria established by Statistics Canada (Canada, 2022); and (3) lived in the Province of Ontario.

### Procedure

#### Surveys

Using Qualtrics, we delivered three surveys beginning 2 months following the first pandemic lockdown in the Province of Ontario in 2020 (T1 = May–June 2020; T2 = October–November 2020; May–June 2021). Only participants who completed surveys at T1 were contacted to participate in T2–T3 surveys. At the end of the first and third surveys, we asked participants if they would be willing to be contacted to participate in qualitative interviews. Participants in this subsample were purposively selected to obtain a diverse sample based on age, gender, race, income source, and health history. Survey respondents were provided with a $10 gift card or e-transfer for each survey completed across the 1-year data collection period.

#### Qualitative Interviews

**Figure 1.**
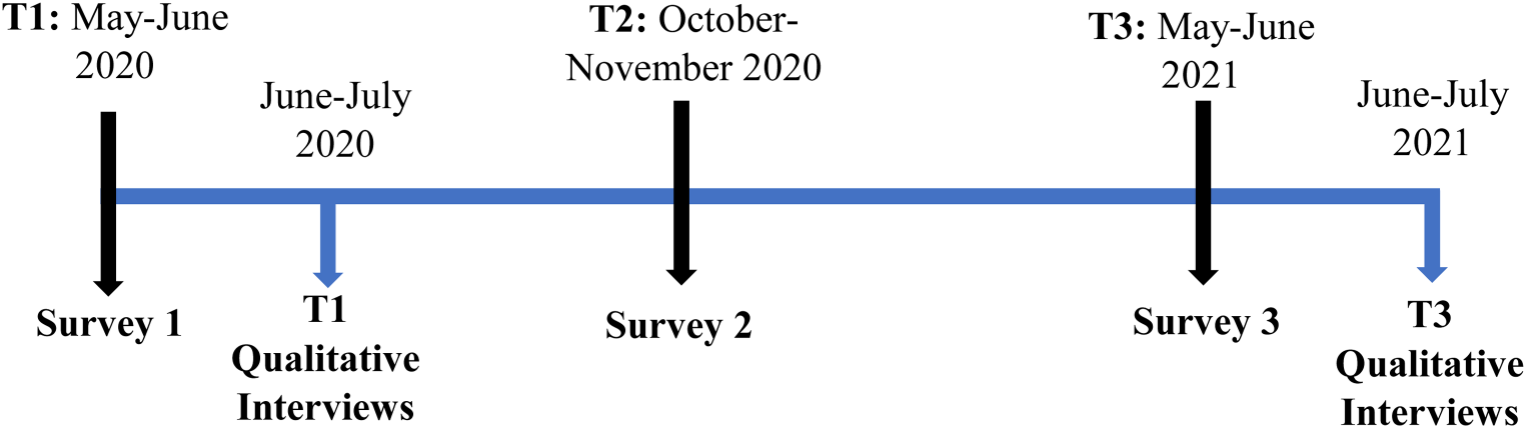
Data collection timeline. ***Note: *** **During T1**, Ontario residents were in the first pandemic lockdown where indoor gatherings of more than five people were restricted and all indoor and outdoor recreational facilities were closed. In addition, private and public elementary and secondary schools were moved to remote delivery. **During T2**, Ontario residents were subjected to easing physical distancing restrictions. Indoor gatherings of more than 10 people, and outdoor gatherings of more than 25 people were restricted. Private and public elementary and secondary schools were delivered in person. Dine-in services were limited to 100 people, and fitness facilities were restricted to 50 people, with no more than 10 people in group classes. **During T3**, Ontario residents were following strict physical distancing restrictions. Outdoor gatherings of up to 10 people were limited, and public and private elementary and secondary schools were moved to remote delivery. Nonessential retailers were restricted to curbside pickup (Canadian Institute for Health Information, 2022).

After completion of T1 surveys, a member of our research team contacted participants using the email address provided in the survey. After completion of an informed consent procedure, we conducted interviews with participants via Zoom or telephone depending upon participant preference. After completion of the T3 survey, participants who had been engaged in qualitative interviews at T1 were approached again to participate in a second qualitative interview. Participants were provided with a $30 gift card or e-transfer for participating in each qualitative interview. See Figure 1 for a visual representation of the timeline for collecting survey data and conducting qualitative interviews.

### Instruments

#### Surveys

****Surveys began with screening questions to determine eligibility including a question that asked participants about their age, and household income and composition. To ensure that only participants living on low incomes participated in our study, we designed our survey so that it excluded participants who reported incomes above the LIM based on the number of individuals living in their household so that we would only include participants meeting the low-income threshold identified by Statistics Canada (2022). Following this, we administered the 96-item survey that included demographic questions (age; gender; sexual orientation; race/ethnicity; community population size; household size; caregiver status; source of income; employment status; health conditions), followed by nine standardized measures exploring two primary constructs (meaningful activity and psychosocial wellbeing). A description of these measures and associated internal consistency scores are detailed in Table 1.

**Table 1:** Description of Standardized Scales and Reliability

Scale	Description	IC in previous research	IC in present study
EMAS	12-item inventory of one's engagement in meaningful activity using a 5-point Likert scale ranging from “never” to “always.” A high score indicates a greater degree of engagement in meaningful activities. This scale measures activity in terms of its subjective meaning to the individual. As such, the meaning in such an activity is defined by the person completing the scale (Goldberg et al., 2002)	α = 0.89 (Goldberg et al., 2002)	α = 0.92
MSBS-8	8-item scale that identifies “state” boredom using a 7-point Likert scale ranging from “strongly disagree” to “strongly agree.” A high score indicates a greater degree of state boredom	α = 0.91 (Oxtoby et al., 2018)	α = 0.92
IMRS (Item 5)	A single question derived from a 15-item measure of recovery for persons living with mental illness. Item 5 asks a participant to identify the amount of time spent in structured or productivity roles on a 5-item ordinal scale from “2 h less per week” to “more than 30 h per week” (Mueser & Gingerich, 2011).	n/a^1^	n/a^1^
PHQ-9	9-item inventory which measures mood on a 4-point Likert scale ranging from “not at all” to “nearly every day.” A high score corresponds with greater degrees of depression	α = 0.86–0.89 (Kroenke et al., 2001)	α = 0.91
GAD-7	7-item scale measuring anxiety on a 4-point Likert scale ranging from “not at all” to “nearly every day.” A high score corresponds with greater degrees of anxiety	α = 0.92 (Spitzer et al., 2006)	α = 0.93
BHP	2-item, positively worded measure of hopelessness on a 5-point Likert scale ranging from “absolutely agree” to “absolutely disagree.” A high score indicates a greater degree of hopelessness	Intraclass correlation coefficient = 0.72 (Fraser et al., 2014)	α = 0.85
UCLA-LS	20-item scale measuring loneliness on a 4-point Likert scale ranging from “I often feel this way” to “I never feel this way.” A high score indicates a higher degree of loneliness	α = 0.96 (Russell et al., 1980)	α = 0.96
AUDIT-10	10-item inventory using a 3–5-point nominal scale corresponding to an established score related to severity of alcohol use. A high score indicates greater use of alcohol	α = 0.75–0.97 in previous research (Reinert & Allen, 2007)	α = 0.69^2^
DAST-10	10-item dichotomous scale (YES/NO) that assesses the extent of a person's substance use. A high score indicates greater degree of drug misuse	α = 0.86 (Cocco & Carey, 1998)	α = 0.74

^1^ This measure includes only one item, and therefore was not included in an assessment of internal consistency.

^2^ In the “questionable” range for internal consistency according to **George and Mallery (2003)**.

Abbreviations: AUDIT-10= Alcohol Use Disorders Identification Test-10; BHP= Brief-H-Pos; DAST-10= Drug Abuse Screening Test-10; EMAS= Engagement in Meaningful Activities Survey; GAD-7= Generalized Anxiety Disorders Scale; IC= internal consistency; MSBS-8= Multidimensional State Boredom Scale-8; IMRS= Illness Management and Recovery Scale; PHQ-9= Personal Health Questionnaire-9; UCLA-LS= UCLA Loneliness Scale.

#### Qualitative Interviews

**Figure 2.**
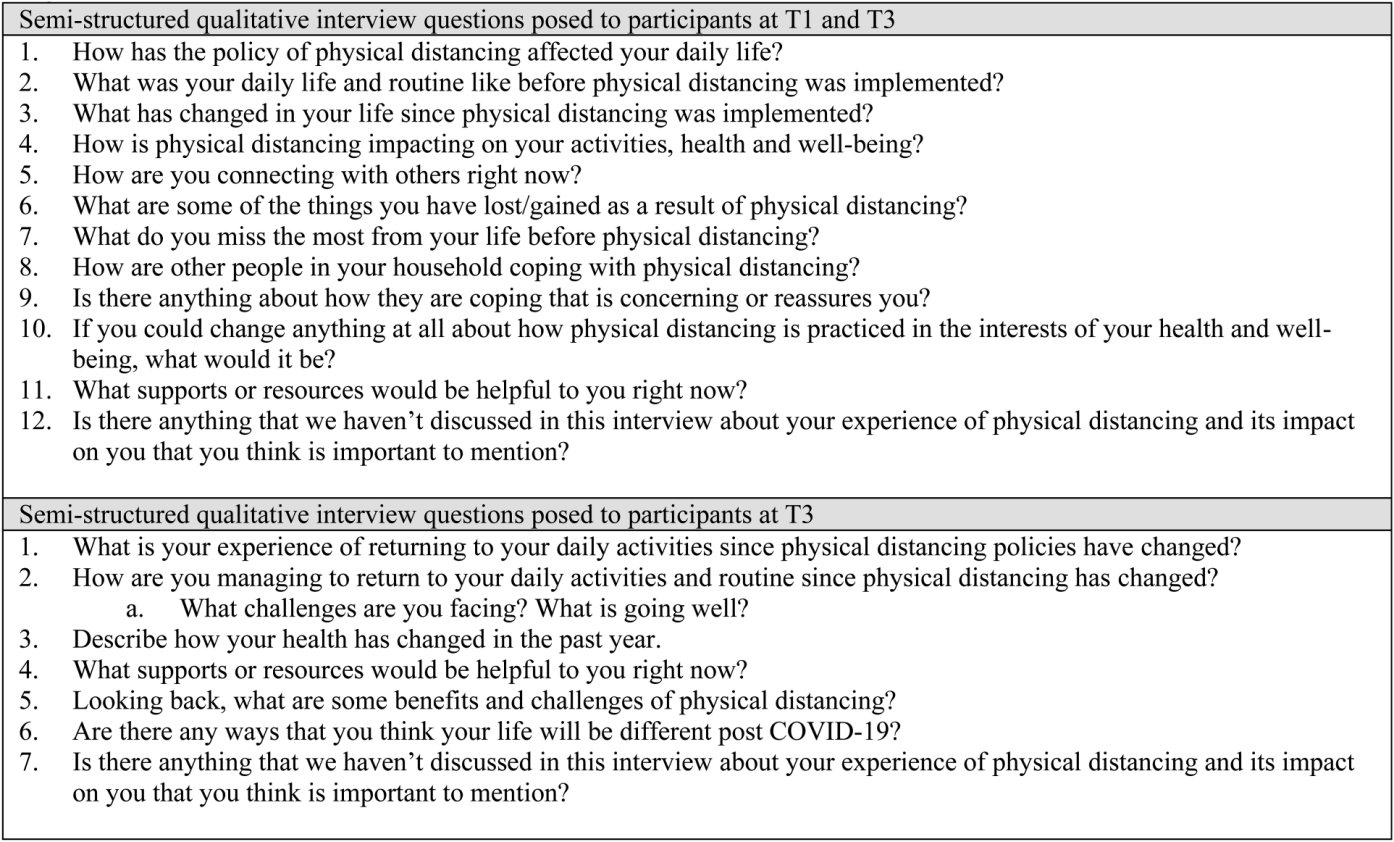
Semi-structured interview questions at T1 and T3.

Qualitative interviews were semistructured and focused on experiences of physical distancing and how they influenced participants’ daily lives, including meaningful activity. The first interview (following T1) focused on experiences of physical distancing policies, and how these influenced daily life and the wellbeing of participants. The second interview (following T3) included the same questions but focused on how physical distancing influenced daily life including meaningful activity over the previous year (see Figure 2). A sample of qualitative interview questions delivered at T1 and T3 is provided in Figure 2.

### Analysis

#### Quantitative

Using SPSS (v. 28), we calculated descriptive statistics for all variables. Demographic characteristics were calculated for the full participant sample at T1. These were calculated again for participants at T2 and T3 to describe the sample composition across all three time points. We conducted one-way analysis of variances (ANOVAs) and Chi-square tests to identify any significant differences in demographic characteristics across T1–T3 groups to understand whether attrition could be attributed to demographic factors. We used Fisher's exact tests when the expected cell counts were less than 5. Summary scores were generated for each standardized measure using processes described by the test authors. As our data were ordinal and not normally distributed, we opted to use nonparametric statistics.

To identify associations between indices of meaningful activity engagement (Engagement in Meaningful Activities Survey [EMAS], Multidimensional State Boredom Scale-8 [MSBS-8], IMRS-5) and psychosocial wellbeing (Personal Health Questionnaire-9 [PHQ-9]; Generalized Anxiety Disorders Scale 7 [GAD-7]; Brief H-Pos; UCLA Loneliness Scale [UCLA-LS]; Alcohol Use Disorders Identification Test-10 [AUDIT-10]; Drug Abuse Screening Test-10 [DAST-10]), we conducted Spearman correlations for data collected from T1 to T3. To compare any differences across these time points, we also conducted Friedman tests. To gain insight into any significant differences, we conducted post-hoc Mann–Whitney U tests. To minimize type 1 error, we used a Bonferroni correction to account for multiple Mann–Whitney U tests by adjusting our minimum significance level to .017 instead of the standard .05 typically used in social and health sciences research (Field, 2013) by dividing .05 by 3 to account for three tests. With the exception of post-hoc tests, minimum significance was set to *p *< .05.

#### Qualitative

T1 and T3 transcripts were separated and uploaded to Dedoose, a qualitative data management program, to facilitate analysis (SocioCultural Research Consultants, LLC, 2015). T1 and T3 transcripts were analyzed separately to enable our team to describe distinct experiences across two time points. Transcripts were coded abductively, informed by intersectionality theory by several members of our research team (JH, SA, BP, CAM, and RG). Using thematic analysis (Braun & Clarke, 2014), our team met on several occasions to arrange codes into themes. Following recommendations of Braun and Clarke (2014), we identified an overarching essence that captured experiences of physical distancing early (T1) and later (T3) in the pandemic.

#### Trustworthiness

Trustworthiness was established using criteria described by Lincoln and Guba (1985) including (1) prolonged engagement with the population of interest, established by our research team's extensive involvement in research and practice related to poverty; (2) peer debriefing among our research team during the conduct of interviews, and in the process of analyzing our data; (3) recording interviews; (4) accurate transcription; (5) intercoder consensus (see analysis); and (6) use of a computer program to organize, sort, and code the qualitative data.

#### Reflexivity

****Collectively, our research team has decades of experience in research and practice with individuals who live in poverty, and several members of our team are occupational therapists. Our extensive involvement in research and practice in this area has informed the design of this study, and how we have analyzed our qualitative data. We have embraced this knowledge and believe that these background experiences have enabled us to analyze the narratives of participants with greater depth.

## Findings

### Sample Characteristics

A total of 108 participants completed surveys at T1, n = 66 at T2, and n = 64 at T3 representing an overall attrition rate of 40.8% from T1 to T3. Across T1–T3, a greater proportion of participants identified as 2SLGBTQ+ (9.4%) at T3 than at T1 (6.5%) and T2 (6.1%). There were also some significant differences in the proportion of participants who were employed at T1 (20.4%) versus T2 (13.6%) and T3 (15.6%). A smaller proportion of participants relied on workers’ compensation benefits (income support provided to individuals injured at work) at baseline (11.1%) versus T2 (18.1%) and T3 (18.8%), and a greater proportion relied on employment insurance (income support provided to individuals who have unexpectedly lost a job) at T3 (9.4%) versus T1 (6.5%) and T2 (6.5%). There were no other demographic differences in participants across T1–T3. A complete summary of the demographic characteristics of participants at T1–T3 is provided in Table 2.

**Table 2:** Participant Characteristics at Times 1–3

Characteristic	Time 1 (n = 108)	Time 2 (n = 66)	Time 3 (n = 64)	*p*-value
	n (%)	n (%)		
Age	Mdn = 43; IQR = 21; 19–72	Mdn = 44; IQR = 25; 24–72	Mdn = 44; IQR = 23; 22–67	.483^1^
≥19–24	4 (3.7)	1 (1.5)	2 (3.1)	
≥25–54	79 (73.1)	45 (68.2)	47 (73.4)	
≥55	25 (23.1)	18 (27.3)	15 (23.4)	
Gender				.390^2^
Man	36 (33.3)	23 (34.8)	24 (37.5)	
Woman	68 (63.0)	42 (63.6)	38 (59.4)	
Non-binary	4 (3.7)	1 (1.5)	2 (3.1)	
Race/ethnicity				.457^2^
White	79 (73.1)	47 (71.2)	43 (67.2)	
Black	9 (8.3)	6 (9.1)	6 (9.4)	
Asian	6 (5.6)	4 (6.1)	3 (4.7)	
Indigenous, First Nations, Inuit or Metis	4 (3.7)	2 (3.0)	4 (6.3)	
Hispanic	1 (0.9)	1 (1.5)	1 (1.6)	
Mixed race	3 (2.8)	1 (1.5)	2 (3.1)	
Other	6 (5.6)	5 (7.6)	5 (7.8)	
Do you identify as 2SLGBTQ+?				
Yes	7 (6.5)	4 (6.1)	6 (9.4)	.042*^2^
No	31 (28.7)	22 (33.3)	18 (28.1)	
Prefer not to answer	3 (2.8)	1 (1.5)	0 (0)	
Missing	67 (62.0)	39 (59.1)	40 (62.5)	
Do you have any physical, cognitive, or mental health conditions?				
Mental health	57 (52.8)	34 (51.5)	31 (48.4)	.329^3^
Physical	55 (50.9)	38 (57.6)	33 (51.6)	.074^3^
Cognitive	11 (10.2)	8 (12.1)	7 (10.9)	.528^3^
None	27 (25.0)	18 (27.3)	17 (26.6)	.651^3^
What is your primary source of income?				
ODSP	50 (46.3)	34 (51.5)	31 (48.4)	.236^3^
OW	25 (23.1)	15 (22.7)	15 (23.4)	.811^3^
CPP	6 (5.6)	5 (7.6)	3 (4.7)	.405^2^
Employment	22 (20.4)	9 (13.6)	10 (15.6)	.022*^3^
Worker's compensation (WSIB)	12 (11.1)	12 (18.2)	12 (18.8)	.001**^2^
Self-employment	7 (6.5)	4 (3.7)	4 (6.3)	1.00^2^
EI	7 (6.5)	7 (6.5)	6 (9.4)	.043*^2^
CERB	4 (3.7)	4 (3.7)	3 (4.7)	.295^3^
OAS	2 (1.9)	2 (3.0)	1 (1.6)	.525^2^
GIS	2 (1.9)	2 (3.0)	1 (1.6)	.525^2^
Short-term disability (employer paid)	1 (0.9)	1 (1.5)	1 (1.6)	1.00^2^
Student loans and grants	1 (0.9)	0 (0)	0 (0)	.380^2^
Alternative employment (e.g., panhandling, sex work, etc.)	3 (2.8)	3 (4.5)	6 (9.4)	.052^2^
Other	9 (8.3)	3 (4.5)	6 (9.4)	.080^2^

*Note:* Percentages do not all equal 100 due to rounding.

*Note:* Only the smallest *p*-values calculated on ANOVA, Chi-square, and Fisher's exact tests comparing the proportion of participants in each category for T1–T3 groups are reported.

^1^ One-way ANOVA.

^2^ Fisher's exact.

^3^ Chi-square.

*p < .05; **p < .01.

Abbreviations: ANOVA= analysis of variance; CERB=Canada Emergency Response Benefit; CPP=Canada Pension Plan; EI=Employment Insurance; GIS=Guaranteed Income Supplement; IQR=interquartile range; Mdn= median; OAS=Old Age Security; ODSP= Ontario Disability Support Program; OW= Ontario Works; WSIB= Workplace Safety and Insurance Board; 2SLGBTQ+ = Two-Spirit, Lesbian, Gay, Bi-Sexual, Trans, Queer or Other Non-Heterosexual Orientation.

In terms of residential and family characteristics, a greater proportion of participants at T1 reported living in a rural community (n = 10; 9.3%) than at T2 (n = 2; 3.0%) and T3 (n = 2; 3.1%). A complete summary of the residential and family characteristics of participants at T1–T3 is provided in Table 3.

**Table 3:** Residential and Family Characteristics at Times 1–3

Characteristic	Time 1 (n = 111)	Time 2 (n = 66)	Time 3 (n = 64)	*p*-value
	n (%)	n (%)	n (%)	
Where do you live?				.701^1^
In a home that I rent at market value	46 (42.6)	28 (42.4)	27 (42.2)	
Social housing	25 (23.1)	15 (22.7)	16 (25.0)	
In a home that I own	22 (20.4)	14 (21.2)	14 (21.9)	
In a home where I don’t pay rent	6 (5.6)	3 (4.5)	3 (4.7)	
In an emergency shelter	1 (0.9)	0 (0)	0 (0)	
Prefer not to say	3 (2.8)	2 (3.0)	2 (3.1)	
Other	7 (6.3)	4 (6.0)	2 (3.1)	
What is the population size of the community in which you reside?				.030*^1^
Rural	10 (9.3)	2 (3.0)	2 (3.1)	
<30,000	10 (9.3)	5 (7.5)	8 (12.5)	
30,000–99,999	20 (18.5)	14 (21.2)	13 (20.3)	
100,000–499,999	33 (30.6)	20 (30.3)	19 (29.7)	
>500,000	35 (32.4)	25 (37.9)	22 (34.4)	
How many people live in your household?				.198^1^
1	50 (46.3)	35 (53.0)	34 (53.1)	
2	20 (18.5)	11 (16.7)	8 (12.5)	
3	18 (16.7)	10 (15.2)	12 (18.8)	
4	15 (13.9)	8 (12.1)	8 (12.5)	
5	4 (3.7)	2 (3.0)	2 (3.1)	
6	1 (0.9)	0 (0)	0 (0)	
How many children under age 18 do you care for?				.150^1^
0	69 (63.9)	45 (68.2)	44 (68.8)	
1	19 (17.6)	11 (16.7)	9 (14.1)	
2	11 (10.2)	5 (7.5)	8 (12.5)	
3	5 (4.6)	4 (6.0)	3 (4.7)	
4	3 (2.8)	0 (0)	0 (0)	
Are you the primary caregiver of a person with a disability?				.489^2^
Yes	26 (24.1)	17 (25.8)	17 (26.6)	
No	82 (75.9)	49 (74.2)	47 (73.4)	

*Note:* Percentages do not all equal 100 due to rounding.

*Note:* Only the smallest *p*-values calculated on Chi-square and Fisher's exact tests comparing the proportion of participants in each category for T1-T3 groups are reported.

^1^ Fisher's exact.

^2^ Chi-square.

*p < .05.

### Quantitative Findings

#### Correlations Between Indices of Meaningful Activity Engagement and Psychosocial Wellbeing Across T1-T3

Across T1–T3, there were medium to large correlations between indices of meaningful activity engagement (EMAS, MSBS-8, and IMRS-5) and indices of psychosocial wellbeing (PHQ-9, GAD-7, Brief H-Pos, and UCLA-LS). Of note, higher EMAS scores and lower levels of boredom (MSBS-8) were significantly correlated with better psychosocial wellbeing overall across all three time points with the majority at the *p* < .01 significance level. One exception to this was that higher EMAS and lower MSBS scores were significantly associated with decreased UCLA-LS scores at T1; however, the opposite association was observed at T2 and T3. Further, indices of meaningful activity engagement (EMAS, MSBS-8, and IMRS-5) were only significantly correlated with alcohol use (AUDIT-10) at T2. Drug use (DAST-10) was not significantly correlated with indices of meaningful activity engagement across T1–T3, which was likely due to the low levels of drug use reported by participants in the sample overall. See Table 4 for a detailed correlation matrix representing data across T1–T3.

**Table 4:** Correlations (Spearman) Between Indices of Meaningful Activity and Psychosocial Wellbeing

			Indices of psychosocial wellbeing					
	MSBS	IMRS-5	Brief-H-Pos	UCLA-LS	GAD-7	PHQ-9	AUDIT-10	DAST-10
**Indices of meaningful activity**								
**Time 1 (n = 108)**								
Meaningful activity engagement (EMAS)	−.475**	.151	−.559**	−.491**	−.401**	−.566**	−.026	−.024
Boredom (MSBS-8)	-	.094	.429**	.543**	.643**	.605**	.078	.080
Productivity time (IMRS-5)		-	−.028	.013	.108	−.024	.108	.081
								
**Time 2 (n = 66)**								
Meaningful activity engagement (EMAS)	−.615**	.313*	−.625**	.568**	−.423**	−.588**	−.332**	−.233
Boredom (MSBS-8)	-	−.230	.456**	−.591**	.495**	.573**	.302*	.120
Productivity time (IMRS-5)		-	−.226	.132	−.005	−.259*	−.346**	−.130
								
**Time 3 (n = 64)**								
Meaningful activity engagement (EMAS)	−.622**	.178	−.739**	.570**	−.499**	−.631**	−.223	.178
Boredom (MSBS-8)	-	−.228	.576**	−.650**	.620**	.645**	.233	−.228
Productivity time (IMRS-5)		-	−.328**	.197	−.160	−.259*	−.006	−.177

*Note:* Using criteria provided by **Cohen (1988)**, a small correlation ranges from .10 to .29, a medium correlation from .30 to .40, and a large correlation from .5 to 1.0.

*p < .05; **p < .01.

Abbreviations: AUDIT-10= Alcohol Use Disorders Identification Test-10; BHP= Brief-H-Pos; DAST-10= Drug Abuse Screening Test-10; EMAS= Engagement in Meaningful Activities Survey; GAD-7= Generalized Anxiety Disorders Scale; MSBS-8= Multidimensional State Boredom Scale-8; PHQ-9= Personal Health Questionnaire-9; UCLA-LS= UCLA Loneliness Scale.

#### Differences in Indices of Meaningful Activity Engagement and Psychosocial Wellbeing Across T1–T3

Complete data from T1 to T3 were available for 47 to 50 participants of the full sample for all standardized measures. This is presented as a range because while 50 individuals participated across T1–T3, not all participants completed standardized measures in full. Because Friedman tests are sensitive to missing data and require data from all time points, only participants for whom complete data was available were included in our analyses. Friedman tests conducted to identify differences in indices of meaningful activity engagement (EMAS, IMRS, and MSBS-8) and psychosocial wellbeing (PHQ-9, GAD-7, Brief H-Pos, UCLA-LS, AUDIT-10, and DAST-10) revealed significant differences across T1–T3. Descriptive statistics and the results of Friedman tests, post-hoc tests, and effect sizes are summarized in Table 5.

**Table 5:** Friedman Tests Identifying Differences in Boredom, Meaningful Activity and Indices of Psychosocial Wellbeing Across Times 1–3 with Post-Hoc Tests

Construct	n	T1 Mdn (IQR)	T2 Mdn (IQR)	T3 Mdn (IQR)	X^2^	*p* (2-tailed)^a^	Post-hoc tests with effect sizes								
							Wilcoxon Signed-Rank tests						Effect sizes (r)		
							T1–T2		T2–T3		T1–T3		T1–T2	T2–T3	T1–T3
Meaningful activity							**Z**	***p* (2-tailed)**	**Z**	***p* (2-tailed)**	**Z**	***p* (2-tailed)**			
EMAS	49	46 (14)	46 (17)	45 (13)	9.110	.011*	−.018	.986	−3.375	<.001***	−2.609	.009**	-	.34^d^	.26^c^
IMRS-5	47	3 (4)	3 (3)	3 (2)	8.89	.012*	−.580	.562	−2.271	.023^b^	1.473	.141	-	-	-
MSBS-8	50	33.5 (23)	34 (24)	36 (24)	1.206	.547									
															
Psychosocial Wellbeing															
PHQ-9	49	6 (11)	18 (13)	11 (13)	66.521	<.001***	−5.664	<.001***	−5.707	<.001***	1.681	.093	.57^e^	.58^e^	-
GAD-7	49	7 (12)	17 (12)	7 (11)	63.174	<.001***	−6.050	<.001***	−5.767	<.001***	.000	1.00	.61^e^	.58^e^	-
Brief H-Pos	49	5 (5)	5 (4)	4 (3)	4.133	.127									
UCLA-LS	49	25 (28)	57 (34)	37 (37)	31.388	<.001***	4.427	<.001***	−6.037	<.001***	−.893	.372	.45^d^	.61^e^	-
AUDIT-10	49	1 (3)	0 (11)	3 (8)	41.067	<.001***	2.995	.003**	4.305	<.001***	−5.778	<.001***	.30^d^	.43^d^	.58^e^
DAST-10	49	0 (0)	0 (0)	0 (0)	3.893	.143									

^a^ Friedman Test p-values: * *p* < .05; **p < .01; ***p < .001.

^b^ To minimize type-1 error, we have used a Bonferroni correction to account for multiple Wilcoxon Signed-Rank Tests. As such, minimum significance for post-hoc tests has been set to .017 instead of .05 to account for 3 distinct tests (i.e., .05 divided by 3).

^c^ Corresponds with a small effect size according to Cohen (1988).

^d^ Corresponds with a medium effect size according to Cohen (1988).

^e^ Corresponds with a large effect size according to Cohen (1988).

Abbreviations: AUDIT-10= Alcohol Use Disorders Identification Test-10; BHP= Brief-H-Pos; DAST-10= Drug Abuse Screening Test-10; EMAS= Engagement in Meaningful Activities Survey; GAD-7= Generalized Anxiety Disorders Scale; IQR= interquartile range; Mdn = median; MSBS-8= Multidimensional State Boredom Scale-8; PHQ-9= Personal Health Questionnaire-9; T1 = Time 1; T2 = Time 2; T3 = Time 3; UCLA-LS= UCLA Loneliness Scale.

##### Meaningful Activity

EMAS scores were significantly different across time points [X^2^ (2, n = 49) = 9.110, *p* < .05]. Inspection of the median values showed a small decrease in EMAS scores from T1 (median [Mdn] = 46; interquartile range [IQR] = 14) and T2 (Mdn = 46; IQR = 17) to T3 (Mdn = 45; IQR = 13). Post-hoc tests confirm that EMAS scores decreased significantly from T2 to T3 (z = −3.375, *p* < .001) and from T1 to T3 (z = −2.609, *p* < .01) both with medium effect sizes (r = .34 and r = .26, respectively). IMRS-5 (productivity time) scores decreased significantly across time points [X^2^ (2, n = 47) = 8.89, *p* < .05]. Median values, however, remained the same across T1 to T3 [T1 (Mdn = 3; IQR = 4); T3 (Mdn = 3; IQR = 2)]. Post-hoc tests revealed a decrease from T2 to T3, but this decrease was nonsignificant at *p* < .017 level (z = −2.271, *p* = .023). MSBS-8 (boredom) increased from T1 to T3; however, this increase was not statistically significant [X^2^ (2, n = 50) = 1.206, *p* = .547]. These findings indicate that engagement in meaningful activity decreased slightly over the course of the first year of the pandemic, and that this decrease occurred mostly from T2 to T3. Further, productivity time and boredom did not significantly increase or decrease over the first year of the COVID-19 pandemic even though meaningful activity decreased over time.

##### Psychosocial Wellbeing

 PHQ-9 (depression) scores were significantly different across time points [X^2^ (2, n = 49) = 66.521, *p* < .001]. Inspection of the median values showed an increase in PHQ-9 scores from T1 (Mdn = 6; IQR = 11) to T2 (Mdn = 18; IQR = 13) and a decrease from T2 to T3 (Mdn = 11; IQR = 13). Post-hoc tests confirm that PHQ-9 scores increased significantly from T1 to T2 (z = −5.664, *p* < .001) and decreased significantly from T2 to T3 (z = −5.707, *p* < .001) both with large effect sizes (r = .61 and r = .58, respectively). There were no statistically significant changes in PHQ-9 scores overall from T1 to T3. Similarly, GAD-7 (anxiety) scores were significantly different across T1 to T3 [X^2^ (2, n = 49) = 63.174, *p* < .001]. Inspection of the median values showed an increase in anxiety at T2 (Mdn = 17; IQR = 12), while these values were the same at T1 and T3. Post-hoc tests confirm that GAD-7 scores increased significantly from T1 to T2 (z = −6.050, *p* < .001) and decreased significantly from T2 to T3 (z = −5.767, *p* < .001) both with large effect sizes (r = .61 and r = .58, respectively). UCLA-LS scores were significantly different across T1 to T3 [X^2^ (2, n = 49) = 31.388, *p* < .001]. Median values increased from T1 (Mdn = 25; IQR = 28) to T2 (Mdn = 57; IQR = 34) and then decreased from T2 to T3 (Mdn = 37; IQR = 37). Post-hoc tests confirm that UCLA-LS scores increased significantly from T1 to T2 (z = 4.427, *p* < .001) and decreased significantly from T2 to T3 (z = −6.037, *p* < .001) both with large effect sizes (r = .45 and r = .61, respectively). Brief H-Pos (hopelessness) scores were not significantly different across T1 to T3 [X^2^ (2, n = 49) = 4.133, *p* = .127]. These findings suggest that there was a similar pattern for depression (PHQ-9), anxiety (GAD-7), and loneliness (UCLA-LS) in that all increased significantly at T2, falling back to baseline levels by T3.

AUDIT-10 (alcohol use) scores were significantly different across T1 to T3 [X^2^ (2, n = 49) = 41.067, *p* < .001]. Inspection of the median values showed a decrease in median AUDIT-10 scores from T1 (Mdn = 1; IQR = 3) to T2 (Mdn = 0; IQR = 11) and an increase at T3 (Mdn = 3; IQR = 8). Post-hoc tests revealed a significant median difference from T1 to T2 (z = 2.995, *p* < .01), T2 to T3 (z = 4.305, *p* < .001) and a significant increase from T1 to T3 (z = −5.778, *p* < .001) with medium to large effect sizes (r = .30, r = .43, and r = .58, respectively). DAST-10 (drug use) scores were not significantly different across T1–T3 [X^2^ (2, n = 49) = 3.893, *p* = .143]. While alcohol use increased over T1 to T3, it should be noted that a score of “8” or higher on the AUDIT-10 is considered to be a “hazardous” level of drinking according to the test authors (Babor et al., 2001), and a median score of three falls well below this threshold. These findings suggest that neither alcohol nor drug use increased or decreased to a level of concern over the first year of the COVID-19 pandemic for participants in this study.

### Qualitative Findings

#### Participant Characteristics

****A total of 27 participants from the T1 surveys engaged in qualitative interviews. Of these, 14 participated at T3. With respect to gender, participants interviewed at T1 identified as: women (n = 18; 66.7%); men (n = 5; 18.5%); nonbinary (n = 3; 11.1%); and two-spirit (n = 1; 3.7%). With regard to race and ethnicity, most participants at T1 were White (n = 19; 70.4%); however, some identified as Asian (n = 3; 11.1%), Hispanic (n = 1; 3.7%), Black (n = 1; 3.7%), and Indigenous (n = 1; 3.7%). Most T1 participants reported that the Ontario Disability Support Program (ODSP) was their primary source of income (n = 20; 74.1%), followed by the Canada Emergency Response Benefit (CERB) (an emergency income support fund provided to individuals who lost work related to COVID-19 pandemic, offered between April 2020 and May 2022) (n = 1; 3.7%), workers’ compensation (n = 1; 3.7%) and other (n = 5; 18.5%). All but n = 2 (7.4%) T1 participants reported living with a health condition including physical health (n = 19; 70.4%), mental health (n = 16; 59.3%), and cognitive health (n = 3; 11.1%) conditions.

#### Overarching Essence (T1): Physical Distancing Exacerbating Exclusion

The essence of our analysis at T1 was that physical distancing protocols served to exacerbate exclusion from meaningful activity that persons living in low income already faced prior to the pandemic. This was expressed through three themes generated in our analysis: (1) limitations on time use entrenching inequities; (2) striving for meaning; and (3) feeling imprisoned in one's home.

##### Limitations on Time Use Entrenching Inequities

Overall, participants at T1 described how physical distancing policies prevented them from engaging in necessary daily activities that promoted wellbeing: “*I can’t go to the gym anymore. That helped my mental health*” [Sharon]. Activities that they performed with ease prior to the pandemic were made more difficult as they tried to avoid contracting the COVID-19 virus. Participants noted that they could not afford to have groceries delivered, and that having to go out to stores placed their health at risk more than individuals who could afford grocery delivery: “*I have to get groceries. I have to eat…so it's venturing out that's quite stressful for me because not everybody is careful*” [Sharon]. The experience of this was described by participants as a sense of loss: “*I’ve lost creative time*” [Stephen]; “*I’ve lost social time, exercise time, and mental health time*” [Elaine]. Many described how the presence of physical distancing policies, which were described by participants as “ableist,” were responsible for creating or entrenching disability: “*Quarantine has very much affected the way that disabled people get around the world*” [Thomas]. The loss of amenities that enabled participants to engage in meaningful activities easily in the community before the pandemic were no longer available due to physical distancing policies and led to ever-increasing isolation: “*I would love for them to figure out how I could take public transit. I would love to be able to go to places*” [Tova].

##### Striving for Meaning

The loss of activities that were necessary for participants to engage in previously were longed after by participants. In their absence, participants sought to find meaning in what they could do while following physical distancing protocols. Many were driven to engage in productivity activities specifically, as having fewer opportunities to contribute engendered negative impacts on mental wellbeing: “*I have not been able to feel like I’m really contributing much of anything at all…and that's not particularly good for my mental well-being” [Dara]*. Some participants expressed that having additional free time enabled them to slow down and finish projects that were challenging to complete prior to the onset of the pandemic: “*I have time to just stay home and not be running around like a chicken with my head cut off*” [Adelita]. Others used unoccupied time to learn new hobbies or reconnect with activities that were meaningful to them in the past, but in which they had been unable to engage for a long time: [I am] “*trying to read books for fun because I have a bit more time, and I haven’t read books for fun in about five years*” [Tova].

##### Feeling Imprisoned in One's Home

Severe limitations on participants’ time use caused some to feel like they had been imprisoned: “*I feel like I’m in jail because I’m in my room by myself*” [Lee]; “*I feel like I’m tethered on a short leash, and I don’t like it*” [Michelle]. These changes in routines came on suddenly: “*it's like night and day. A complete change*” [John]. The suddenness with which physical distancing policies were implemented caused an immediate halt and suspension of regular activities. This, combined with supply chain issues, made it feel to participants like Sharon like the end of the world:

This feels like an apocalypse…Like when I went into the grocery store the first time and seen the bare shelves, I cried…I couldn’t wrap my head around how I couldn’t buy my kids toilet paper…like “oh my God. We’re gonna run out of diapers.” [Sharon]

Some participants lived with health conditions that increased vulnerability to contracting the virus and experiencing complications associated with COVID-19. This caused them to isolate in their apartments, without seeing anyone or leaving to perform necessary activities:

It has extremely affected my life. I am disabled and immunocompromised…it extremely affected my mental health when we first went into quarantine. So I went into extreme lockdown in my own apartment. I didn’t even leave to do laundry. I don’t have a balcony that's useable. [Thomas]

##### Overarching Essence (T3): COVID as a Layer of Burden on Everything I Do

The essence of our analysis of T3 qualitative interviews was that after the pandemic wore on over time, participants felt a sense that physical distancing protocols added an extra layer of burden that made everything more difficult than it was before. These narratives were particularly powerful as most participants already lived with disabilities that challenged them in their daily lives prior to the onset of the pandemic, and ongoing changes to physical distancing restrictions created confusion and generated additional emotional and cognitive burdens. This essence was expressed through three themes generated in our analysis: (1) finding meaning through relationships with activity; (2) COVID-19 has magnified the inequities I live with; and (3) Classist and ableist pandemic policies have made daily life so much harder.

##### Finding Meaning Through Relationship With Activity

As the year progressed, participants endured a range of physical distancing restrictions that ebbed and flowed as COVID-19 case numbers fluctuated and public health officials responded. Physical distancing rules evolved constantly, causing confusion for participants about how they could spend their time in community spaces. Participants had different experiences over the year, but most continued to struggle to fill time in meaningful ways and maintaining a sense balance in activities continued to be a problem: “*my life was really busy before…I didn’t always take time for myself. And now I have too much time for myself*” [Elaine]. Having little to do, however, continued to motivate participants to find new activities to fill time. They saw activities as a way of coping by “weathering” the storm of COVID-19: “*I bought a set of bongos, which I’ve always wanted to do, and started to teach myself a little bit*” [Karen]; “*I do a lot more meditation and mindfulness than I ever have*” [Stuart].

##### COVID has Magnified the Inequities I Live With

Participants discussed how living in low income was an additional barrier that made it challenging to cope with physical distancing restrictions imposed during the first year of the pandemic. They indicated that an inadequate income forced them to make decisions when performing daily activities that placed them at greater risk of contracting COVID-19 than individuals living on more adequate incomes: “*I can’t afford to have groceries delivered. That means that I have to go out*” [Elaine]. Participants indicated that this situation was compounded by the fact that the cost of food and other necessary resources had increased: “*a lot of things have gone up*” [Karen]. However, despite record levels of inflation driving up the cost of basic needs, income support benefits had not followed suit, and placed participants in ever-increasing precarity: “*I just go further and further in debt each month*” [Lynn]. Participants recognized that they needed to perform activities differently to respond to growing income inequality, and they expressed frustration over this challenging situation: “*How can you have a functioning society when so many people are hanging on by their fingernails like this? It's madness*” [Dara].

##### Classist and Ableist Pandemic Policies Have Made Daily Life so Much Harder

As the pandemic wore on, participants described how there seemed to be no end in sight, and physical distancing policies made everything they did with their time more emotionally and cognitively taxing. As many were living with disabilities prior to the pandemic, activities were already more difficult to perform, and participants noted that physical distancing policies failed to account for their specific needs, making them feel as though their needs simply did not matter. They emphasized that this tiered system existed prior to the pandemic: “*my life has not really been impacted by social distancing restrictions, it's been impacted by the fact that we are treated like…dogs to be put down at worst*” [Jeane]. This point was emphasized as they compared the introduction of the CERB, a benefit available to individuals who had lost employment due to the pandemic. The amount for CERB was set to $2,000/month, while funding allotted to individuals living on the ODSP (disability related social assistance) was set to approximately $1,200/month, and Ontario Works (OW, general social assistance) at just over $600/month: “*when they decided last year that $2,000 was the amount for people that were off work from the pandemic. We get half that. It's just a slap in the face*” [Karen]. The poverty in which participants lived as they observed this growing inequality restricted their ability to function in daily life to such an extent that it caused them to consider opting for medical assistance in dying:

You know how many people I know who are thinking of taking medical assistance in dying because they’re living on the same ODSP income that they’ve always lived on? $1,169 a month. And they live alone. And they can’t get any more money, and everyone else who lost money or lost a job got $2,000 a month each. [Lynn]

Further, some participants highlighted that the challenges faced by persons living in low income and with disabilities during the pandemic were unacceptable and hoped that inequities revealed during the COVID-19 experience would lead to needed changes after the pandemic ended: “*If anything good comes out of the COVID pandemic, it will be the fact that we’re now more willing to question those basic assumptions to a greater extent than we were before. Because ‘normal’ wasn’t working*” [Dara].

## Discussion

We conducted this study to explore: (1) how individuals living in low income experienced meaningful activity during the first year of the COVID-19 pandemic; (2) how indices of meaningful activity engagement and psychosocial wellbeing were associated; and (3) meaningful activity and psychosocial wellbeing evolved during this time period. Our findings reveal that physical distancing policies, which have been necessary for controlling the spread of the COVID-19 virus, have imposed unintended, negative influences on the ability of persons living in low income to participate in meaningful activity and attain psychosocial wellbeing. Approximately 75% of participants in our study identified as living with health conditions, and most participants in our qualitative sample identified as living with disabilities. As they reflected back on experiences over the first year of the pandemic, they expressed anger and frustration that public health policies failed to account for their specific needs. They continued to live in poverty as the prices of goods and services increased and income support rates did not. Participants had limited opportunities for meaningful activity, and this meant that they were less likely to experience improvements in psychosocial wellbeing as greater engagement in meaningful activity and lower boredom were associated across all three time points with decreased hopelessness, anxiety, and depression. Over half of participants in both the survey and qualitative interviews identified as women, and from an intersectional (Crenshaw, 2017) lens, these findings emphasize how intersections of income, disability, and gender converged in the first year of the pandemic to exclude participants from meaningful activity that was essential for supporting psychosocial wellbeing.

Our quantitative findings reveal that indices of meaningful activity engagement were positively associated with several measures of psychosocial wellbeing over the first year of the pandemic and that meaningful activity decreased over time. Participants confirmed these findings in qualitative interviews and indicated that access to meaningful activity was limited in their lives both by living in poverty and the need to follow physical distancing protocols. Psychosocial wellbeing declined significantly at T2, and meaningful activity engagement followed. This decrease in psychosocial wellbeing has been observed in other studies exploring the mental health impacts of the COVID-19 pandemic for a range of populations (Boden et al., 2021; Khan et al., 2022). We can only theorize as to why increases in meaningful activity were associated with decreased loneliness at T1, yet associated with increased loneliness at T2 and T3 in our correlational analyses, and why psychosocial wellbeing increased at T3 after such a significant decline at T2 especially in light of the fact that pandemic restrictions were more strict at T3 than T2. Our qualitative findings provide a glimpse into why these patterns may have emerged. While participants experienced extreme isolation and a range of challenges to participating in meaningful activity early in the pandemic, that over time, they employed creative strategies to increase engagement in meaningful activity in order to cope with the severe restrictions imposed by public health regulations. With respect to loneliness, participants may have acknowledged the fact that they were experiencing loneliness at T2 and T3, and accepted that they, and others in society, were experiencing the same phenomenon. Thus, psychological reappraisal (Uusberg et al., 2019) of participants’ circumstances may have occurred, resulted in a pattern of increased psychosocial wellbeing at T2, despite acknowledgement that opportunities for meaningful activity had lessened and loneliness was more present in their lives. In essence, it is possible that they became more psychologically resilient (Fletcher & Sarkar, 2013) over time as they adapted to pandemic restrictions. To confirm these theories, however, future studies that explore adaptation to physical distancing restrictions are needed.

### Research and Practice Implications

Our findings reinforce the findings of recent existing research indicating that meaningful activity and psychosocial wellbeing are closely associated, and that individuals have faced barriers to meaningful activity participation during the COVID-19 pandemic (Cruyt et al., 2021; Tigershtrom & Boyraz, 2021; Wegner et al., 2022). While scholars have indicated that meaningful activity could be an antidote to the stress and other psychosocial challenges faced by individuals during the COVID-19 pandemic (Boden et al., 2021), only a few studies have been conducted in this area that focus specifically on meaningful activity (Cruyt et al., 2021; Tigershtrom & Boyraz, 2021; Wegner et al., 2022). As our research was longitudinal and conducted in a Canadian context, this study builds on existing research by exploring the ways in which meaningful activities evolved over the first year of the COVID-19 pandemic in Canada. Further, this study is the only known that has focused specifically on meaningful activity and psychosocial wellbeing of persons living in low income during this time.

The findings of this research underscore the critical importance of meaningful activity for psychosocial wellbeing. Occupational therapists and occupational therapy researchers should work together to explore these relationships further, with a particular focus on influencing policy, and developing and evaluating interventions tailored to meeting the activity needs of persons limited by low incomes as public health policies continue to evolve. Co-designing interventions alongside persons with lived experience may provide opportunities for developing approaches that are relevant and feasible for persons living in poverty (Zamenopoulos & Alexiou, 2018). Researchers and practitioners may consider developing such approaches at the individual, community, and population level in the current and future pandemics to mitigate barriers to accessing meaningful activity and attaining psychosocial wellbeing imposed by physical distancing policies (Michie & West, 2021).

### Policy Implications

The findings of this research emphasize the ways in which necessary public health policies that help limit the spread of disease can impose other unintended consequences for human health. While we did not intend for our research to focus specifically on disability, the high rates of poverty among persons living with disabilities resulted in a sample that lived with a high prevalence of health conditions, and most participants in our qualitative sample identified as disabled. As such, they faced barriers to engagement in meaningful activity prior to the pandemic, and physical distancing restrictions imposed an additional layer that limited participation. Our research emphasizes the need for public policy that mitigates the unintended impacts of physical distancing on the meaningful activities of persons who experience both poverty and disability to reduce harms that limiting engagement in meaningful activity may cause. Allocating funding for public health initiatives that support the implementation of programs that address barriers to meaningful activity engagement during pandemic restrictions need to be employed in concert with emergency measures. Policymakers may consider consulting both with persons with lived experiences of poverty and occupational therapists in the development of such policies (Kamalakannan & Chakraborty, 2020). Finally, the importance of increasing income support program rates, or introducing a basic income cannot be overstated. Advocates have long called for reform (Haagh, 2019), and increasing the household income among persons living in poverty may both increase their ability to participate in meaningful activities, as well as perform activities in a way that lessens exposure to COVID-19.

### Limitations

This research was conducted with individuals living in poverty in one province in a high-income country. Sample sizes in our quantitative analyses were small given the high rate of attrition over the three data collection periods in this study. In terms of race, gender, and sexual orientation, participants in this study were mostly White, more than 60% were women or nonbinary, and few identified as 2SLGBTQ + . Further, most of the participants in our qualitative sample were women, and as such, the reader should be aware that our qualitative findings reflect a distinctly feminine perspective. The composition of our samples should be accounted for when interpreting our findings, and we recommend that future research be undertaken with more diverse samples with respect to race, gender, and sexual orientation.

## Conclusion

During the time of preparing this article, the pandemic is continuing to unfold. While physical distancing restrictions have recently been lifted in Ontario, Canada, the risk of contracting the COVID-19 virus continues to fluctuate (Province of Ontario, 2022). Scholars predict that future pandemics are on the horizon (Kamalakannan & Chakraborty, 2020; Khan et al., 2022). The findings of this study and previous research highlight important associations between meaningful activity engagement and psychosocial wellbeing during the first year of the COVID-19 pandemic (Cruyt et al., 2021; Tigershtrom & Boyraz, 2021; Wegner et al., 2022). The findings presented in this article build on these existing studies by emphasizing the particular impacts of physical distancing restrictions on the meaningful activity engagement and psychosocial wellbeing of persons living in poverty. Participants in this study faced barriers to participating in meaningful activity during the first year of the COVID-19 pandemic due to intersections of poverty and disability. Attending to these associations is critical for informing strategies designed to mitigate negative psychosocial impacts of physical distancing policies for disadvantaged populations.
